# Assessment of DNA-PKcs kinase activity by quantum dot–based microarray

**DOI:** 10.1038/s41598-018-29256-2

**Published:** 2018-07-20

**Authors:** Florian Lafont, Nizar Ayadi, Cathy Charlier, Pierre Weigel, Igor Nabiev, Houda Benhelli-Mokrani, Fabrice Fleury

**Affiliations:** 1 Group of Mechanism and Regulation of DNA Repair and IMPACT platform, UFIP UMR CNRS 6286/University of Nantes, 44322 Nantes, France; 2 Laboratoire de Recherche en Nanosciences, LRN-EA4682, UFR de Pharmacie, Université de Reims Champagne-Ardenne, 51100 Reims, France; 3 Laboratory of Nano-Bioengineering, National Research Nuclear University MEPhI (Moscow Engineering Physics Institute), 115409 Moscow, Russian Federation

## Abstract

Therapeutic efficacy against cancer is often based on a variety of DNA lesions, including DNA double-strand breaks (DSBs) which are repaired by homologous recombination and non-homologous end joining (NHEJ) pathways. In the past decade, the functions of the DNA repair proteins have been described as a potential mechanism of resistance in tumor cells. Therefore, the DNA repair proteins have become targets to improve the efficacy of anticancer therapy. Given the central role of DNA-PKcs in NHEJ, the therapeutic efficacy of targeting DNA-PKcs is frequently described as a strategy to prevent repair of treatment-induced DNA damage in cancer cells. The screening of a new inhibitor acting as a sensitizer requires the development of a high-throughput tool in order to identify and assess the most effective molecule. Here, we describe the elaboration of an antibody microarray dedicated to the NHEJ pathway that we used to evaluate the DNA-PKcs kinase activity in response to DNA damage. By combining a protein microarray with Quantum-Dot detection, we show that it is possible to follow the modification of phosphoproteomic cellular profiles induced by inhibitors during the response to DNA damage. Finally, we discuss the promising tool for screening kinase inhibitors and targeting DSB repair to improve cancer treatment.

## Introduction

Understanding cellular systems requires identification and analysis of the functions of cellular components, especially their functional interrelation and regulation. In response to DNA damage, active cell signaling pathways initiate a series of protein phosphorylation cascades. These processes involve a network of complex interactions of cellular components that coordinate the DNA Damage Response (DDR)^1^. Studying the DDR requires a comprehensive approach involving analysis of a large set of parameters related to modifications of cellular phosphoproteome.

Among DNA lesions, DSBs are the most toxic. They can be repaired by two major mechanisms, Homologous Recombination (HR) and Non Homologous End Joining (NHEJ), wherein Rad51 and DNA-PKcs, respectively, play a pivotal role^1^.

The DNA-PKcs kinase is essential for the NHEJ DNA repair pathway, and is involved in several biological processes, such as mitosis^2^, protection of telomeres^3^, and maturation of the immune system^4^. DNA-PKcs belongs to phosphatidylinositol-3 kinase-like kinase family (PIKK) including ATM (ataxia-telangiectasia mutated) and ATR (Rad3-related protein), which are key components for the detection and signaling of DNA damage. C-region of DNA-PKcs contains a kinase domain, which is involved in its auto-phosphorylation and the phosphorylation of other proteins after DNA damage^2,5^. Among the substrate proteins in the DNA repair machinery, replication protein A (RPA) is recognized as one of the major proteins and is multiphosphorylated by DNA-PKcs, in particular its subunit RPA2^6^. Additionally, the DNA-PKcs kinase has been demonstrated to play a critical role in the development of chemoresistance^7^. Indeed, several groups have investigated the level and activity of DNA-PKcs in tumors and have suggested their correlation with the resistance and malignant properties of cancer cells^8–11^. Moreover, several investigations have shown that inhibition of DNA-PKcs by small molecules radiosensitises or chemosensitises cancers, such as osteosarcoma, glioma, breast, lung, and colon cancer models^7,12–15^. Thus, the development and screening of DNA-PKcs kinase inhibitors might improve the efficacy of the current cancer treatments inducing DNA damage.

In recent decades, the microarray technology has become one of the few tools providing excellent results for this type of analysis^16^. One advantage of the protein microarray technology is the possibility of highly rapid and sensitive high-throughput recognition and analysis of multiple targets. It has the potential to evaluate, in a single experiment, the protein level and the post-translational modification by phosphorylation in a sample from a cellular fraction, biopsy, or biological fluid (e.g., serum)^17^. The antibody microarrays approach allows the analysis and comparison of the proteome or phosphoproteome of normal cells and cancer cells. Today, detection of microarray signals usually employs organic fluorophores, which often suffer from low sensitivity and instability due to their photodegradation. Recent data show that the use of highly fluorescent semiconductor nanocrystals or quantum dots (QDs) is a promising alternative^17–19^. Indeed, the spectral characteristics of QDs are exceptional: in addition to a great stability, they have a high brightness with a quantum yield reaching 100% and high extinction coefficients in the UV and visible regions of the optical spectrum^20,21^. Furthermore, the spectral positions of the emission bands of different QDs widely vary, the peak wavelength depending on the QD composition and diameter (400 nm to 2 μm), whereas the fluorescence emission spectral width of each QD type is narrow^17,19^.

Here, we have developed a QD-based antibody microarray for detection of cell protein phosphorylation changes produced by camptothecin-induced DNA damages. We have demonstrated that, after treatment of cells with the anticancer drug camptothecin (CPT), the phosphorylation level of several DNA repair proteins is strongly increased, and these variations may be quantitatively monitored using the microarray approach. We have demonstrated that the combination of kinase inhibitors with CPT treatment affects the phosphorylation profile in response to DNA damage.

Our results pave the way to preclinical validation of the QD-based microarray approach to screening kinase inhibitors, which is of special interest for pharmaceutical companies.

## Results

### Generation of the antibody microarray dedicated to the NHEJ DNA repair pathway

**Figure 1:**
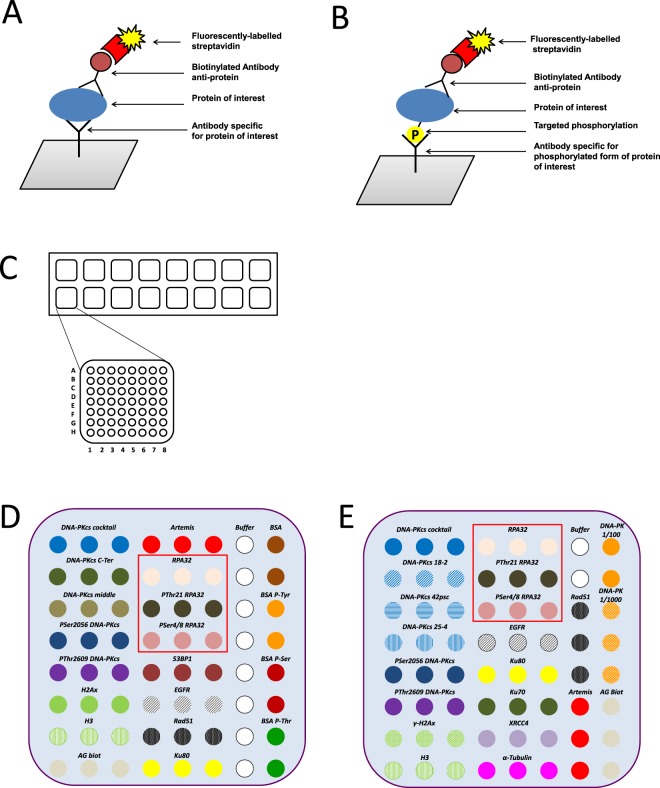
Schematic representation of the antibody microarray experiment. The principle of antibody arrays using fluorophore-streptavidin is described for analysis of protein level (**A**) or for analysis of phosphorylation level of one residue on targeted protein (**B**). Schematic overview of slide (format 16 pads) including the description of one pad for coordinate antibody (**C**). The composition of the antibody microarrays generated from the lot number 1 (**D**) and from the lot number 2 (**E**). Red frame corresponds to anti-RPA2 and anti-phosphorylated RPA2 antibodies spotted in triplicate.

16 antibodies and 4 control proteins were deposited in triplicate on the nitrocellulose membrane pad (Fig. 1). Each slide contains 16 nitrocellulose pads (Fig. 1C). Two lots of home-made microarrays were generated (microarray lot number 1 - Fig. 1D and lot number 2 - 1E); a detailed list of antibodies is shown in Tables 1 and 2 wherein coordinates are specified according to the Fig. 1B. The selection and the specific recognition evaluation of each antibody were performed by Western blotting analysis.

**Table 1:** The list of antibodies deposited and included in the microarray lot number 1 described in the Fig. 1D and used in the Fig. 3A.

Coordinate	Antibody or protein sample
A01-A03	anti-DNA-PKcs_cocktail
B01-03	anti-DNA-PKcs_C-Terminal domain
C01-03	anti-DNA-PKcs_middle
D01-03	anti-PhosphoSer2056
E01-03	anti-PhosphoThr2609
F01-03	anti-H2AX
G01-03	anti-H3
H01-03	anti-goat-biot diluted
A04-06	anti-Artemis
B04-06	anti-RPA2_m
C04-06	anti-PhosphoT21_RPA2
D04-06	anti-PhosphoS4/S8_RPA2
E04-06	anti-53BP1
F04-06	anti-EGFR
G04-06	anti-Rad51
H04-06	anti-Ku80
A07, B07, C07, D07, E07,F07, H07	Tris Saline Buffer
A08-B08	Bovin Serum Albumin (BSA)
C08, D08	BSA_Phospho-Tyr
E08, F08	BSA_Phospho-Ser
F08, G08	BSA_Phospho-Thr

**Table 2:** The list of antibodies deposited and included in the microarray lot number 2 described in the Fig. 1E and used in the Fig. 5A.

Coordinate	Antibody or protein sample
A01-03	anti-DNA-PKcs_cocktail
B01-03	anti-DNA-PKcs_18-2
C01-03	anti-DNA-PKcs_42-psc
D01-03	anti-DNA-PKcs_25-4
E01-03	anti-PhosphoSer2056
F01-03	anti-PhosphoThr2609
G01-03	anti-γH2AX
H01-03	anti-H3
A04-06	anti-RPA2
B04-06	anti-PhosphoS4/S8_RPA2
C04-06	anti-PhosphoT21_RPA2
D04-06	anti-EGFR
E04-06	anti-Ku80
F04-06	anti-Ku70
G04-06	anti-XRCC4
H04-06	anti-αtubulin
A07, B07	Tris Saline Buffer
C07, D07, E07	anti-Rad51
F07, G07, H07	anti-Artemis
A08, B08	DNA-PKcs protein diluted at 1/100
C08, D08, E08	DNA-PKcs protein diluted at 1/10
F08, G08, H08	anti-goat-biotinylated

In our microarray approach, several protein controls were deposited on each pad, to follow different steps of acquisition and detection. Specifically, BSA and phospho-tyrosine-BSA proteins were also spotted as controls to ensure the specific recognition of the anti-phosphotyrosine antibody. Biotinylated protein was spotted to validate the recognition by the streptavidin-fluorophore step (Fig. 1B for coordinate of the antibodies).

### Analysis of DNA-PKcs expression level in the MO59J and MO59K cell lines to compare the detection approaches using AlexaFluor 680 or QDs705

**Figure 2:**
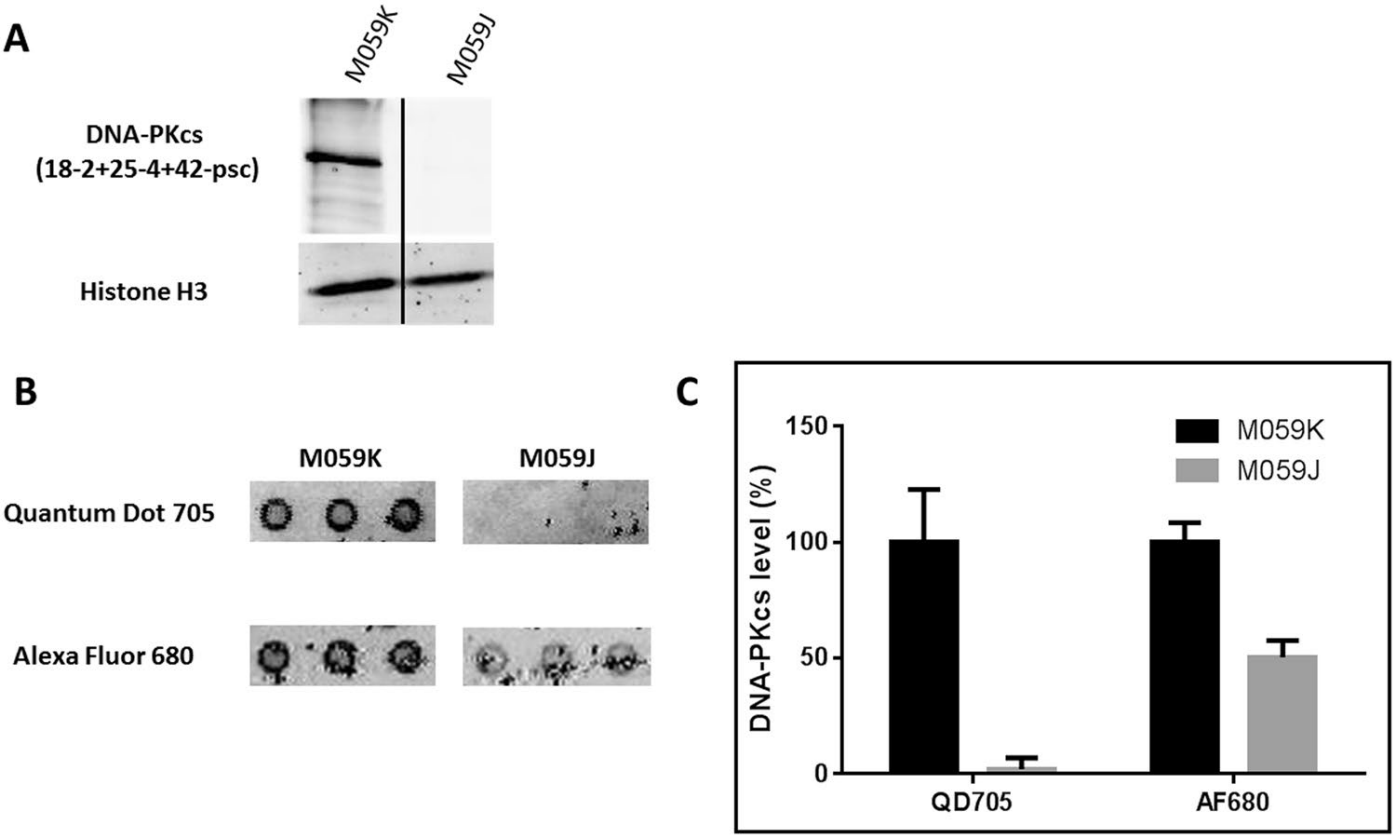
Detection of DNA-PKcs level in glioblastomas by Western blot and the antibody microarray. (**A**) MO59K and MO59J protein extracts were analyzed by immunoblot. They were performed using anti-DNA-PKcs (clone 18− 2+ 25− 4+ 42-psc) and anti-H3 (clone ab1791). (**B**) The same cell extracts were analyzed by the antibody microarray. Briefly, each extract was incubated on 2 pads (from microarray lot number 1, Fig. 1D) and then with an anti-DNA-PKcs (clone 18-2) coupled with biotin. The pads were then developed with streptavidin coupled with QD705 or Alexa Fluor 680. Images were captured with an Odyssey scanner (LI-COR), and (**C**) the signals corresponding to the DNA-PKcs antibody spots (coordinate A01-A03) were quantified with the Genepix software. The signal from the MO59K cell extract was taken to be equal to 100%. The error bars represent standard deviation (SD) of three independent experiments.

MO59J and MO59K are glial cell lines. Together, MO59K and MO59J provide a useful cellular model to study the role of DNA-PKcs in cellular and molecular processes involving DNA damage recognition and repair. MO59K cells express normal levels of DNA-PKcs, while MO59J cells lack DNA-PKcs expression^22–24^. The Western blot analysis confirmed the presence and absence of DNA-PKcs in MO59K and MO59J, respectively (Fig. 2A). A microarray including the DNA-PKcs antibody panel was incubated with the protein extracts of MO59J an MO59K. Retained DNA-PKcs proteins were recognized with biotinylated DNA-PKcs antibody. The revealing was carried out with either QDs-705-streptavidin or AlexaFluor-680-streptavidin. The signal was monitored, and fluorescence intensity of each spot was compared between MO59K and MO59J. Figure 2B shows clearly a signal of DNA-PKcs in MO59K extracts. However in MO59J extract, an unexpected signal appeared only with AlexaFluor but not with QDs. All signals were quantified; Fig. 2C shows the comparison of the fluorescence intensity between both streptavidin-fluorophore complexes. Hence, the analysis on MO59J by using QD-streptavidin detection clearly demonstrates the absence of the DNA-PKcs-mediated signal, whereas the use of the AlexaFluor system shows a significant signal equivalent to 50% of that of MO59K.

Because of this AlexaFluor-mediated background, we decided to use the QD-streptavidin system for the subsequent experiments.

### Response to CPT-mediated DNA Damage in HeLa cell line

**Figure 3:**
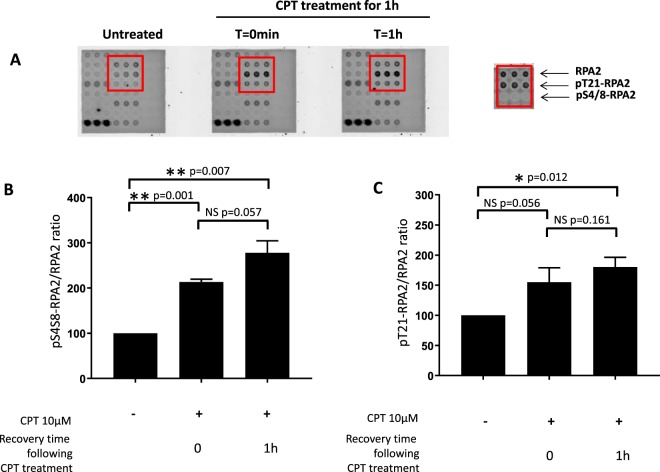
Microarray analysis of RPA2 phosphorylation in HeLa cells in response to CPT treatment. HeLa human cervical epithelial carcinoma cells were treated with 10 µM CPT for 1 h. RPA2 and phospho-RPA2 expression of untreated and treated cells were analyzed by microarray from lot number 1 (**A**) before and after CPT treatment. The phospho-RPA2 to RPA2 signal ratios were determined as described in the Materials and Methods section and were compared with that for the untreated cell extract taken to be 100%. The pSer4/8-RPA2 to RPA2 and pThr21-RPA2 to RPA2 signal ratios are presented in histograms (**B** and **C**, respectively). Data shown are the mean and standard deviation (SD) of three independent experiments. Statistical analysis was performed using paired Student’s t-test (*p < 0.05, **p < 0.01, NS for p > 0.05).

To detect the phosphorylation level of DNA repair proteins, we used the HeLa cell line, which is characterized by a high level of DNA-PKcs^25–28^. The cells were treated with CPT at a final concentration of 10 µM for 1 h, and the level of RPA2 phosphorylation was assessed after different recovery times up to 1 h post-treatment. As shown in Fig. 3, CPT treatment of HeLa cells induced the phosphorylation of RPA2, which is detected and quantified from the microarray analysis as described in the Materials and Methods section. The intensities of the spots corresponding to phosphoRPA2 were strongly increased for Ser4/Ser8 and Thr21 phosphorylated forms of RPA2 (Fig. 3A, red frame), whereas the signals of total RPA2 and control spots were unchanged. The phosphorylation levels were estimated by calculating the phosphoRPA2 to RPA2 ratio and are presented as a histogram (Fig. 3B,C).

**Figure 4:**
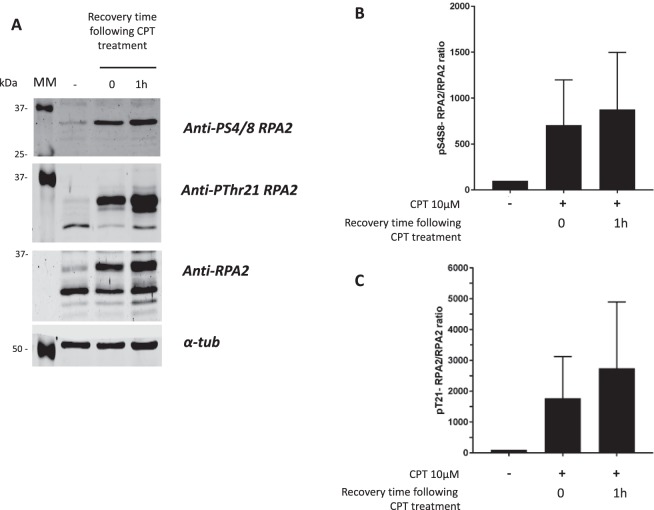
Western blot analysis of RPA2 phosphorylation in HeLa cells in response to CPT treatment. The same extracts of HeLa cells treated with 10 µM CPT for 1 h were analyzed by immunoblotting. Untreated and treated cells were analyzed by immunoblotting (**A**) before and after CPT treatment. The phospho-RPA2 to RPA2 ratios were determined as described in the Materials and Methods section and were compared with that for the untreated cell extract taken to be 100%. The pSer4/8-RPA2 to RPA2 and pThr21-RPA2 to RPA2 ratios are presented in histograms (**B** and **C**, respectively). The error bars represent standard deviation (SD) of two independent experiments.

A clear increase was found in the phosphorylation of RPA2 after CPT treatment and 1 h post-treatment with CPT. This increase was more pronounced for Ser4/Ser8 than Thr21 phosphorylated RPA2 forms (Fig. 3A,B). One hour post-treatment, the phosphorylation ratio continued to increase slightly. To validate this finding, the amounts of different RPA2 forms were assessed using Western blotting (Fig. 4). The treatment of HeLa cells with 10 µM CPT induced several bands with different migration velocities. These bands correspond to the intermediate and hyperphosphorylated statuses of RPA2, which are directly related to DNA-PKcs kinase activity^29,30^.

Anti-RPA2 antibody recognized different forms of RPA2, including the non-phosphorylated and several phosphorylated statuses of RPA2 (Fig. 4A). It is worth noting that the anti-PThr21RPA2 antibody recognizes also the non-phosphorylated RPA2, in contrast to anti-PSer4/8-RPA2 antibody, which seems to be more phospho-specific (Fig. 4A).

As in the previous microarray analysis, the phosphoRPA2 to RPA2 ratio was determined from Western blotting data; the results are presented in Fig. 4B,C. Our findings showed that the ratio was increased after CPT treatment, which was similar for both Ser4/8 and Thr21 phosphorylated RPA2 statuses.

These results show that the microarray enables the assessment of DNA-PKcs activity *via* the RPA2 Ser4/Ser8 and Thr21 phosphorylation statuses, which has been validated by Western immunoblotting analysis.

### Inhibition of DNA-PKcs activity by chemical inhibitors in HeLa cell line

Because the DNA-PKcs activity was described as a potential target in anticancer therapeutic strategy, it was interesting to test some inhibitors of the PIKK protein family by using our microarray approach.

**Figure 5:**
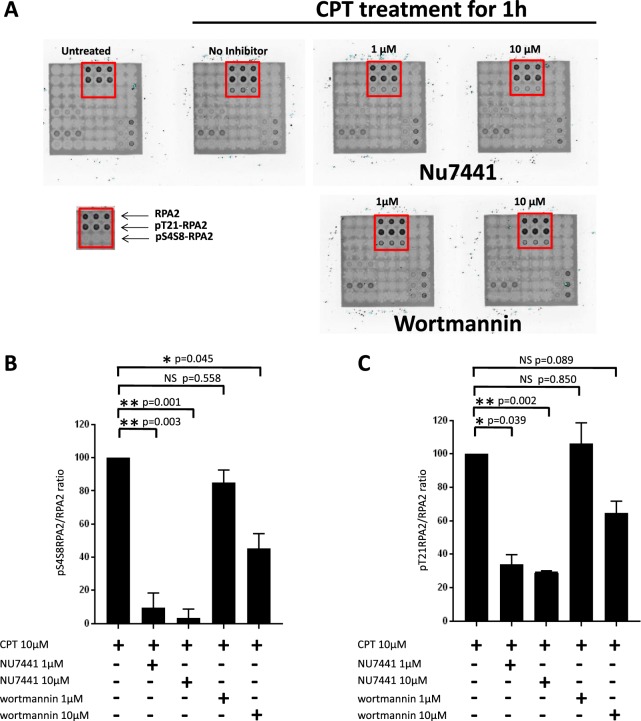
CPT-mediated RPA2 hyperphosphorylation is affected by DNA-PKcs inhibitors. HeLa human cervical epithelial carcinoma cells were pre-incubated with the NU7441 or wortmannin inhibitor at a concentration of 1 or 10 µM for 24 h. Then the cells were treated with 10 µM CPT for 1 h, and cellular extracts were incubated on the microarray from lot number 2 (Fig. 1E). The phospho-RPA2 to RPA2 ratios were determined and compared with that for the CPT-treated cell extract taken to be 100%. The pSer4/8-RPA2 to RPA2 and pThr21-RPA2 to RPA2 ratios are presented in histograms (**B** and **C**, respectively). The phospho-RPA2 to RPA2 ratio of the untreated cell extract was subtracted. Data shown are the mean and standard deviation (SD) of three independent experiments. Statistical analysis was performed using paired Student’s t-test (*p < 0.05, **p < 0.01, NS for p > 0.05).

HeLa cells were pre-incubated with either wortmannin or NU7441 kinase inhibitor for 24 h at increasing concentration before exposure to CPT at 10 µM for 1 h. Thus, the level of RPA2 phosphorylation was assessed in HeLa cells after exposure to CPT combined with pretreatment with kinase inhibitors at a concentration of 1 µM or 10 µM. As expected, an increase in RPA2 phosphorylation was clearly seen after 1 h of CPT treatment for HeLa cells in the absence of a kinase inhibitor (Fig. 5).

This level of RPA2 phosphorylation was clearly different in the case of pretreatment with an inhibitor and depended on the location of the phosphorylation site and the type of inhibitor.

Indeed, the phosphorylation of the RPA2 on Ser4/Ser8 residues is strongly affected by pretreatment with NU7441 at a concentration of 1 µM or higher, with the phosphorylation level decreased by about 90%. This diminution is less pronounced with wortmannin; it was 20% and 55% at 1 and 10 μM, respectively. Finally, for the same concentrations, NU7441 decreases Ser4/Ser8 phosphorylation more strongly in response to CPT than wortmannin.

Regarding the CPT-induced phosphorylation of the Thr21 residue, a significant background level is observed before treatment with CPT (Fig. 5A). However, after subtraction of this background contribution, the treatment with CPT also provokes Thr21 phosphorylation of RPA, which is slightly decreased with both NU7441 and wortmannin pretreatments (Fig. 5C). The Thr21 phosphorylation is unchanged by 1 μM wortmannin, whereas it is inhibited by about 60% upon treatment with NU7441 at the same concentration. At 10 μM, the NU7441 inhibitor induces a phosphorylation decrease two times higher than wortmannin (Fig. 5C).

Overall, we have found that the changes in the CPT-mediated phosphorylation level of RPA2 are less pronounced in the case of wortmannin compared to NU7441, which seems to act in a dose-dependent manner against DNA-PKcs activity (Fig. 5).

**Figure 6:**
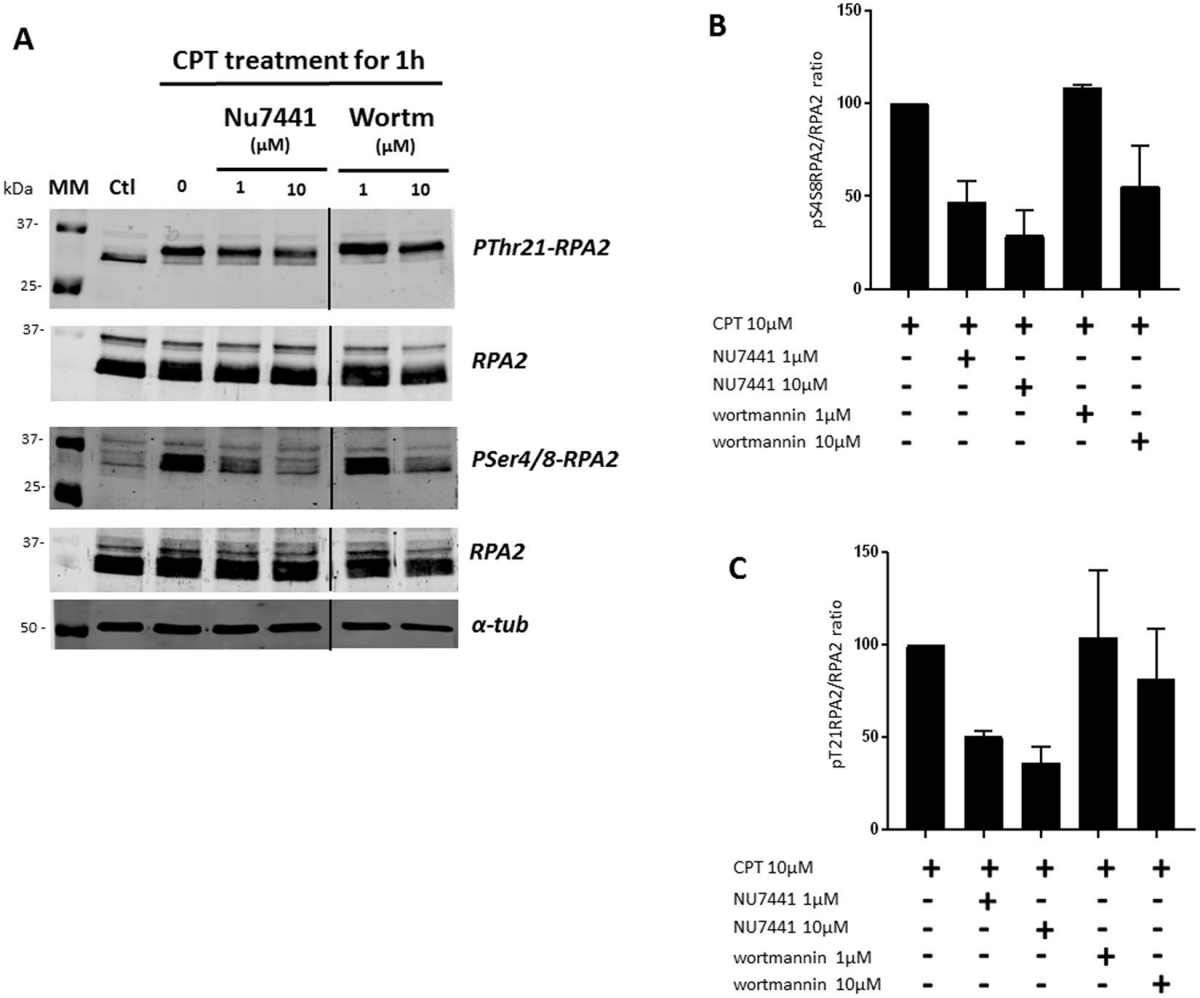
The decrease of CPT-mediated RPA2 hyperphosphorylation is confirmed by immunoblotting analysis. HeLa human cervical epithelial carcinoma cells were pre-incubated with the NU7441 or wortmannin (Wortm) inhibitors at a concentration of 1 or 10 µM for 24 h. Then the cells were treated with 10 µM CPT for 1 h, and cellular extracts were analyzed by immunoblotting (**A**). The phospho-RPA2 to RPA2 ratios were determined and compared with that for the CPT-treated cell extract taken to be 100%. The pSer4/8-RPA2 to RPA2 and pThr21-RPA2 to RPA2 ratios are presented in histograms (**B** and **C**, respectively). The error bars represent standard deviation (SD) of two independent experiments.

The same cellular extracts were then analyzed by Western blotting (Fig. 6).

As was described in the Materials and Methods, we estimated the amounts of phosphorylated RPA2 forms and total RPA2 in order to determine the phosphoRPA2 to total RPA2 ratios, which are presented in Fig. 6B,C. As already shown in Fig. 4 and confirmed in Fig. 6 (with CPT treatment alone), the treatment of HeLa cells with CPT at 10 µM induced an increase in RPA2 phosphorylation. The phosphorylation levels of RPA2 on Ser4/Ser8 (Fig. 6B) and Thr21 (Fig. 6C) are almost similar. Cells pre-incubated with NU7441 and wortmannin exhibited a decrease in RPA2 phosphorylation levels in response to CPT. NU7441 pretreatment results in a decrease of about 50% to 65% in RPA2 phosphorylation levels, while wortmannin causes a 25% decrease at 10 μM.

As microarray analysis has shown, pretreatment with NU7441 induces a more pronounced decrease in RPA2 phosphorylation than wortmannin pretreatment.

This analysis by Western blot confirmed the results obtained with the microarray quantification.

## Discussion

In the past decade, the function of DNA-PKcs has been described as a potential mechanism of resistance in tumor cells. Several studies have shown that NU7441 enhances the cytotoxicity of ionizing radiation (IR) and etoposide in colon cancer cell lines but not in DNA-PKcs deficient cells, indicating that potentiation of DNA damage and cell death is primarily due to DNA-PKcs inhibition^12^. DNA-PKcs has become a potential target in the anticancer strategy, and the development of specific inhibitor of this kinase could improve the treatment by sensitizing tumor cells. For example, it has been shown that the DNA-PKcs inhibitor KU60648 acts as a radiosensitizing agent for osteosarcoma^10^.

CPT is a topoisomerase inhibitor which disrupts the replication process, provoking a replication stress in the cell. Under replicative stresses, RPA2 is hyperphosphorylated at serine 4 and 8 sites (Ser4/Ser8). These RPA2 phosphorylations are essential for the cell to delay mitotic entry and to prevent unscheduled HR at collapsed DNA replication forks^6,31,32^. After camptothecin induced replicative stress, the Ser4/Ser8 hyperphosphorylation of RPA2 is essentially mediated by DNA-PKcs^32,33^.

**Figure 7:**
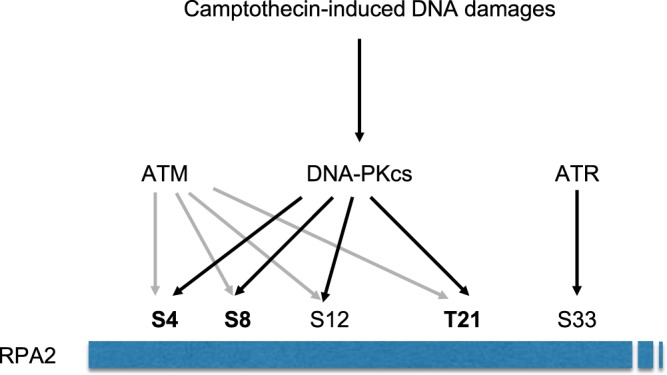
RPA2 Nter domain phosphorylation by PIKKs after CPT induced DNA damages. Depending on the genotoxic treatment used, ATM and DNA-PKcs can share some redundancy in their capacity of phosphorylating RPA2 Nter domain. After CPT induced DNA damages, Ser4, Ser8 and Thr21 phosphorylation are managed by DNA-PKcs and are shown by dark arrows. Gray arrows indicate minor activity of ATM that is not required for CPT induced RPA2 phosphorylation on these same residues^32,33^.

In this study, we developed an antibody microarray that allowed us to assess DNA-PKcs activity after CPT induced DNA damages by following the phosphorylation of its substrate RPA2. We followed Ser4, Ser8 and Thr21 phosphorylations (Fig. 7) to assess DNA-PKcs activity after CPT treatment in the absence and in the presence of two inhibitors. We used the wortmannin compound that inhibits all enzymes of the PI3K family by interacting with their catalytic subunits^34^, and the NU7441 that displays high selectivity for DNA-PKcs and is inactive against both ATM and ATR (>100 µM)^35,36^. Using our antibody microarray we showed that Wortmannin induces a slight inhibition of the DNA-PKcs activity while the NU7441 induces a strongest one. These results, confirmed by a western blot analysis, highlight the ability of this antibody microarray to reveal the different inhibiting properties of two DNA-PKcs inhibitors. That allowed us to consider a high throughput screening of DNA-PKcs possible modulators in the context of the development of new inhibitors of the DNA repair proteins as a therapeutic strategy to prevent the resistance of cancer.

Moreover, by using the antibody arrays, we have shown that it is possible to evaluate different levels of expression and phosphorylation status of DNA repair protein.

Many companies propose antibody microarrays allowing the determination of proteomic and phosphoproteomic profiles of signaling pathways involving cellular factors such as MAPK, Akt, and receptor tyrosine kinases, but there is currently no specific microarray dedicated to DNA repair pathways. This gap in the market can be explained not only by the limited availability of the anti-phospho antibodies, but also by the weak sensitivity of the conventional detection systems (organic fluorophores or chemiluminescence) compared to the detection using inorganic fluorophores, such as QDs. It is noteworthy that, in most commercial systems, the detection of phosphoproteomic levels involves labeling of samples (often through biotinylation), which may lead to additional bias between the samples, in contrast to our sandwich approach (Fig. 1).

Although we have established a proof of concept of the assessment of kinase activity by microarray based on the estimation of the RPA2 substrate phosphorylation, several points remain to be improved. We have chosen nitrocellulose support that has been successfully applied to the generation of protein arrays. Due to the microporous surface of the nitrocellulose membrane, immobilization of numerous proteins is possible with a high signal to noise ratio, which improves the signal detection^37^.

Whereas the cross-recognition of anti-phospho-antibodies is not a limiting factor for Western blot analysis, this feature is crucial for the protein array. We compared anti-phosphoSer4/Ser8 and anti-phosphoThr21 RPA2 and have observed background related to the low specificity of pThr21-RPA2 antibody. The knowledge of the epitope recognized by antibody is also important in our approach. Indeed, the capture epitope should be efficiently different or sufficiently far from the detection epitope in order to avoid the recognition competition between the two antibodies. Our findings have shown this importance of this characteristic by testing several anti-DNA-PKcs antibodies. The signal intensity clearly depends on the distance between the capture epitope and detection epitope. The antibodies choice is thus primordial to ensure the high quality of microarrays.

We used the streptavidin-biotin way to assess and monitor the fluorescence signal. Although the streptavidin-biotin system is simple to set up and use, it does have certain limitations. Biotinylated antibody may indeed bind to any biotin-binding protein from the cell extract, which is the main disadvantage of this streptavidin-biotin system. Because biotin is a biological molecule, endogenous biotin can cause a background signal. To overcome this restriction, the use of fluorophore labeling with a secondary antibody should be an interesting alternative improving the signal to background ratio and removing an additional development step. Moreover, this alternative technique could be used in combination with other detection methods.

The detection by fluorescence has been more and more widely used to monitor signals from protein arrays^37^. This fluorescence detection does not require a particular protection, in contrast to radioactive detection, but depends on the spectroscopical properties of the fluorophores.

In this study, we have shown that the detection by QDs demonstrate lower background signal than that for AlexaFluor dye. Indeed, AlexaFluor seems to interact with protein in a nonspecific manner. In some cases, hydrophobicity of organic fluorescent dyes, such as AlexaFluor, is the main determinant for nonspecific binding^38^.

However, our conditions of monitoring the fluorescence signals were not completely optimized. Indeed, the Li-COR scanner used provided an excitation light not close to the maximal absorption of QDs705. In fact, different types of QDs have been tested, and, as expected, a great number of them were unsuitable in this detection approach. The use of an excitation source in the region of QD first exciton or in the blue-violet region of optical spectrum would improve the signal detection sensitivity, and the development of a detector adapted to the physico-chemical features of QDs would be an improvement for the signal detection with the microarray.

In conclusion, we have reported the feasibility of the QD-based antibody microarray dedicated to DNA damage response.

This tool gives the possibility to evaluate the kinase activity (DNA-PKcs) by measuring the level of phosphorylation of its substrate (RPA2), but it also opens many prospects for analysis of the modifications of the phosphoproteomic profiles of DNA repair pathways, such as the homologous recombination pathway, which involves a succession of phosphorylation cascades at each of its stages^1^, and the NHEJ pathway.

Our study is the first to demonstrate that the microarray can be used to quantify and estimate the efficiency of inhibitors against the DNA-PKcs repair protein target.

## Methods

### Cell culture

MO59J/K and Hela cell lines were purchased from ATCC company (Manassas, Virginia, USA).

The MO59J/K cells were grown in DMEM/F12 medium (Gibco) supplemented with 10% SVF and 5% antibiotics (penicillin, 100 U/ml and streptomycin, 100 µg/ml) under a humidified atmosphere containing 5% CO_2_ at 37 °C.

HeLa cells were maintained in DMEM medium (Gibco) containing 10% FBS and 5% antibiotics (penicillin, 100 U/ml and streptomycin, 100 µg/ml) in a humidified atmosphere with 5% CO_2_ at 37 °C.

### Chemicals

Camptothecin (CPT) and wortmannin were purchased from Sigma (St Louis, MO, USA).

NU7441 was obtained from Euromedex (distributor of SelleckBio products).

The QD-streptavidin conjugate Kit (Thermofisher, ref Q10151MP) contains six conjugates which have a narrow, symmetric emission spectrum with the emission maximum near 525, 565, 585, 605, 655, or 705 nm.

The anti-DNA-PKcs and anti-RPA2 biotinylated antibodies were purchased from Thermo Fisher Scientific (Cell Signaling Technology Waltham, MA, USA).

The anti-phospho(Thr21)RPA2 and anti-phospho(Ser2056)DNA-PKcs antibodies were purchased from Abcam (Cambridge, UK); all others were purchased from Cell Signaling Technology (Danvers, MA, USA).

### Chemical treatment of cells and protein extraction

The cells were seeded at a density of 2∙10^6^ cells per dish.

Cells were pretreated with kinase inhibitors for 24 h and then with 10 µM CPT for 1 h at 37 °C, after which they were washed twice with ice-cold PBS. Cells were lysed in lysis buffer purchased from Cell Signaling Technologies (Danvers, MA, USA) on ice for 20 min under agitation. Cellular extract were sonicated and centrifuged at 10,000 g for 15 min at 4 °C. The soluble fractions were collected, and the protein concentration was determined by BCA assay (Biorad, Hercules, CA, USA).

The samples were either used immediately for microarray assay or stored at −80 °C for immunoblotting analysis.

### Antibody microarray production

Antibodies which specifically target proteins of the NHEJ repair pathway (Tables 1 and 2, Fig. 2) were prepared at concentrations of 0.2–1 mg/ml in phosphate buffer saline in a 384-well microplate (Porvair Sciences, Shepperton, UK). Antibodies of interest were then spotted onto UniSART slides coated with 16 pads of nitrocellulose (Sartorius, Goettingen, Germany) using a sciFLEXARRAYER S3 Piezo Electric Dispenser (Scienion, Berlin, Germany). For all samples, six drops were applied in three replicates onto each pad, and the drop volume was camera controlled (400 pL/drop). The spotting was performed in a chamber at 25 °C and a humidity of 60%. Antibody microarray slides consisted of 16 pads which allowed testing 16 different cellular extracts with a 16-pad hybridization chamber (Whatman, Maidstone, UK). The microarrays were sealed on a slide box and stored at 4 °C until use.

### Incubation of protein extracts on microarray

Before the incubation of cellular extracts, each pad of the microarray was blocked with 5% BSA (w/v) in Tris buffered saline +0.1% Tween 20 (TBS-T) for 2 h at 4 °C with gentle shaking. After three washing steps with TBS-T at RT, each pad was incubated for 2 h at 4 °C with 80 µL of samples containing total protein extracts (with a total protein concentration of 1 mg/ml). Unbound proteins were removed by washing the array for 3 × 5 min with TBS-T.

Incubation with anti-RPA2 or anti-DNA-PKcs biotinylated antibody was performed for 2 h at 4 °C. The slides were washed again and were then incubated for 1 h in the dark with streptavidin-conjugated QDs 705 (4 µg/ml, Thermofisher, ref Q10151MP). The final washing step (3 × 10 min) with TBS-T was done before detecting the fluorescence signal with an Odyssey infrared imaging system scanner at a resolution of 21 µm (LI-COR Biosciences, Lincoln, NE, USA). Light excitation was performed at 685 nm.

### Microarray data analysis

Fluorescence signal intensity was quantified using the GenePix Pro® 4 software (Molecular Devices, Sunnyvale, CA, USA). For each spot, the specific median fluorescence intensity was determined after subtraction of the local background fluorescence intensity. For each pad, the three technical replicates were averaged. Represented values are expressed as the mean percent change from the starting control ± standard deviation. Differences between the means of multiple groups were analyzed using paired Student’s t test for independent samples. Statistical significance was assumed at *p < 0.05, **p < 0.01, or Non-Significant NS for p > 0.05. Statistical analysis and signal values representations were performed using the Graphpad Prism 7 software (GraphPad Software Inc, San Diego, CA).

### SDS-PAGE electrophoresis and immunoblotting

The total cellular extract protein fractions were stored at −80 °C. Equal amounts of cellular lysate proteins (40 μg of total proteins) were separated by electrophoresis on 10% SDS-polyacrylamide gels and transferred to nitrocellulose membranes (GEHealthcare, Little Chalfont, UK). Membranes were blocked for 1 h in 0.1% TBS-Tween 20 containing 2.5% BSA and then incubated with mouse monoclonal antibodies (anti-DNA-PKcs, anti-RPA2, anti-tubulin, and anti-Histone H3) or rabbit antibodies (anti-pRPA2 and anti-pDNA-Pkcs). Protein bands were resolved by fluorescence with anti-mouse AlexaFluor680 or anti-rabbit AlexaFluor680-labelled secondary antibodies (Life Technologies-Invitrogen). The signal intensity (pixels/mm^2^) of protein bands was quantified using the Odyssey software version 1.1 (Li-COR, Biosciences, Lincoln, NE, USA). α-tubulin and H3 were used as a loading control.

## Electronic supplementary material

Supplementary Information
