# Supplementary material for: Assessment of DNA-PKcs kinase activity by quantum dot–based microarray
Source: Sci Rep. 2018 Jul 20;8:10968. doi: 10.1038/s41598-018-29256-2 (PMC6054677; doi:10.1038/s41598-018-29256-2)
Supplement: Supplementary file 1 — Supplementary Information [file 41598_2018_29256_MOESM1_ESM.docx]

**Assessment of DNA-PKcs kinase activity by quantum dot–based microarray**

Florian Lafont^1&^, Nizar Ayadi^1&^, Cathy Charlier^1^, Pierre Weigel^1^, Igor Nabiev^2,3^, Houda Benhelli-Mokrani^1^ and Fabrice Fleury^1,*^

*^1^ Group of Mechanism and Regulation of DNA Repair and IMPACT platform , UFIP UMR CNRS 6286 / University of Nantes, 44322 Nantes, France*

*^2^ Laboratoire de Recherche en Nanosciences, LRN-EA4682, UFR de Pharmacie, Université de Reims Champagne-Ardenne, 51100 Reims, France*

*^3^ Laboratory of Nano-Bioengineering, National Research Nuclear University MEPhI (Moscow Engineering Physics Institute), 115409 Moscow, Russian Federation*

** fleury-f@univ-nantes.fr*

*& F.L. and N.A. contributed equally to this work.*

**
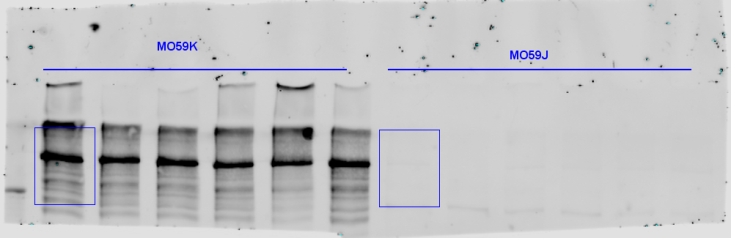
**

***IB :DNA-PKcs***

**A**

**
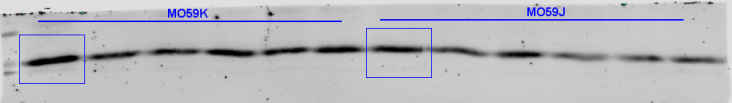
**

***IB : Histone H3***

**B**

**Alexa Fluor 680**

**Quantum Dot 705**

**
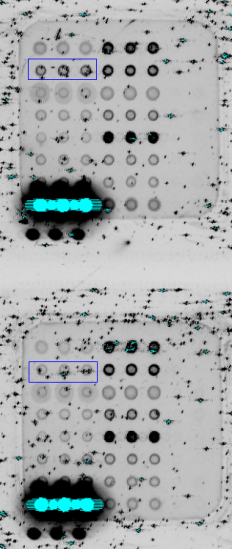

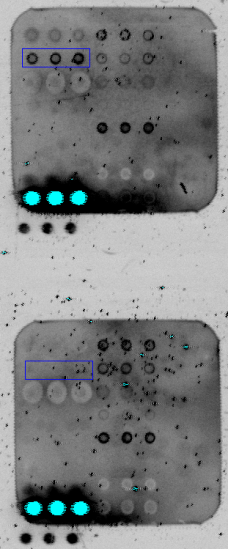
**

**M059K**

**M059J**

**Figure S2. Detection of DNA-PKcs level in glioblastomas by Western blot and the antibody microarray.**

(A) MO59K and MO59J protein extracts were analyzed by immunoblot. They were performed using anti-DNA-PKcs (clone 18-2+25-4+42-psc) and anti-H3 (clone ab1791). (B) The same cell extracts were analyzed by the antibody microarray. The pads were then developed with streptavidin coupled with QD705 or Alexa Fluor 680.


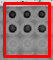

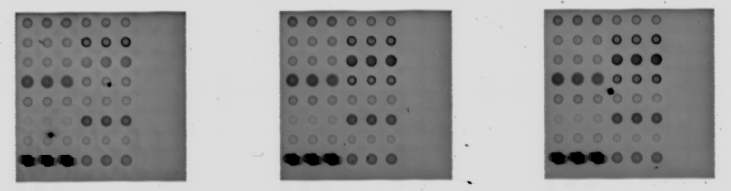


**T=0min**

**T=1h**

**RPA2**

**pT21-RPA2**

**pS4/8-RPA2**

**Untreated**

**CPT treatment for 1h**

**Figure S3. Microarray of RPA2 phosphorylation in HeLa cells in response to CPT treatment.**

HeLa human cervical epithelial carcinoma cells were treated with 10 µM CPT for 1 h. RPA2 and phospho-RPA2 expression of untreated and treated cells were analyzed by microarray from lot number 1(A) before and after CPT treatment.


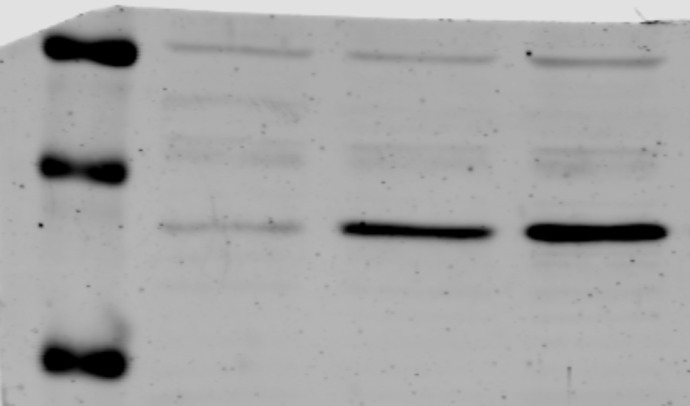


Recovery time following CPT treatment

-

1h

0

kDa MM

37-

***Anti-PS4/8 RPA2***

25-


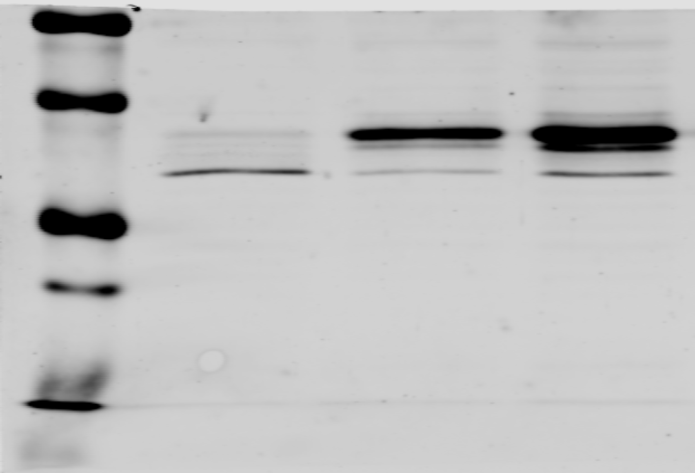


50 -

37-

***Anti-PThr21 RPA2***

**A**

Recovery time

following CPT treatment


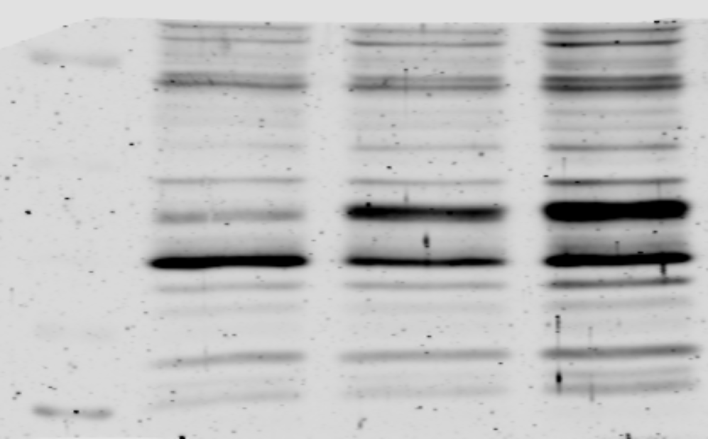


50-

***Anti-RPA2***


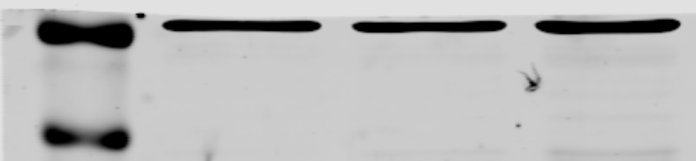


50-

***α-tub***

**Figure S4. Western blot analysis of RPA2 phosphorylation in HeLa cells in response to CPT treatment.** Untreated cells (lane 2) and cells treated with 10 µM CPT for 1 h were analyzed by immunoblotting at t=0h (lane 3) or 1h post-treatment (lane 4). Molecular Marker (MM, lane 1).


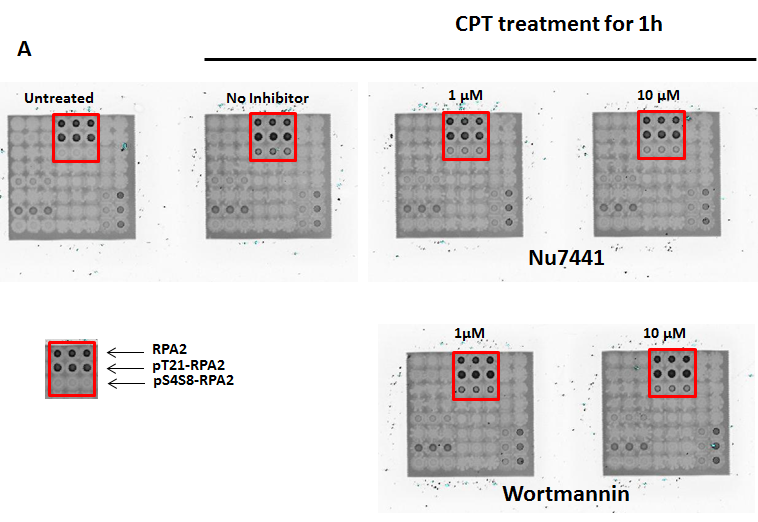


**Figure S5. CPT-mediated RPA2 hyperphosphorylation is affected by DNA-PKcs inhibitors**

HeLa human cervical epithelial carcinoma cells were pre-incubated with the NU7441 or wortmannin inhibitor at a concentration of 1 or 10 µM for 24 h. Then the cells were treated with 10 µM CPT for 1 h, and cellular extracts were incubated on the microarray.

kDa  **MM**

**CPT treatment for 1h**

**Wortm**

**(µM)**

**1**

**10**

**100**

**10**

**0**

**Ctl**

**1**

**Nu7441**

**(µM)**


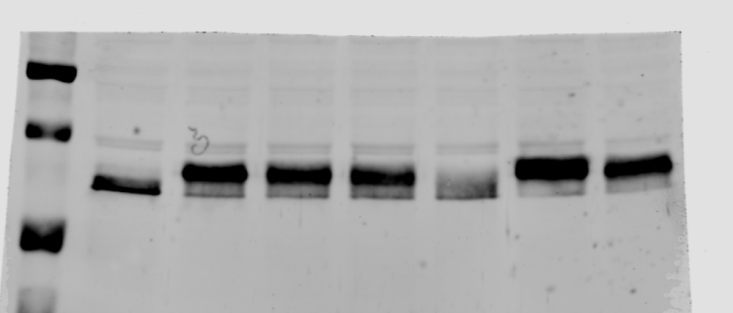


***PThr21-RPA2***

50-

25-

37-


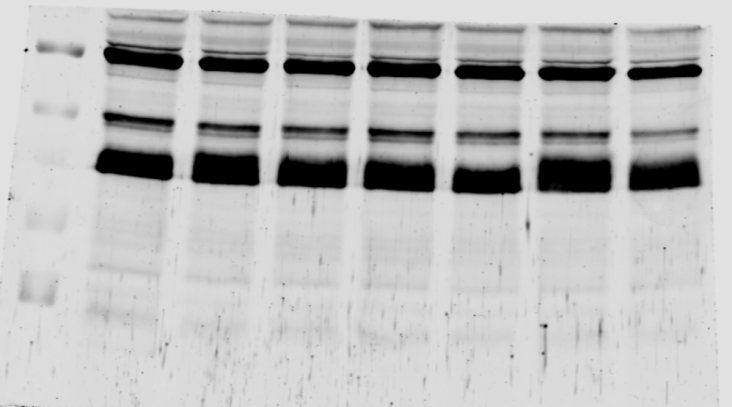


25-

50-

37-

***RPA2***

***α-tub***


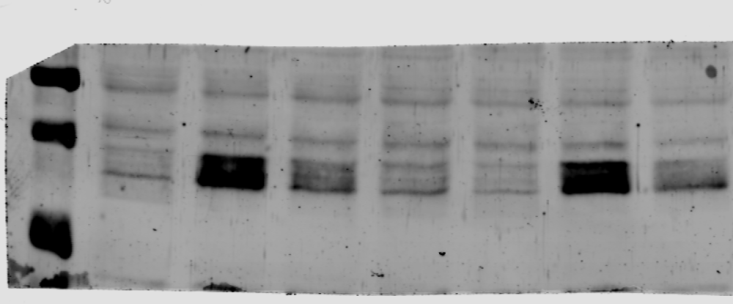


37-

25-

50 -

***PSer4/8-RPA2***


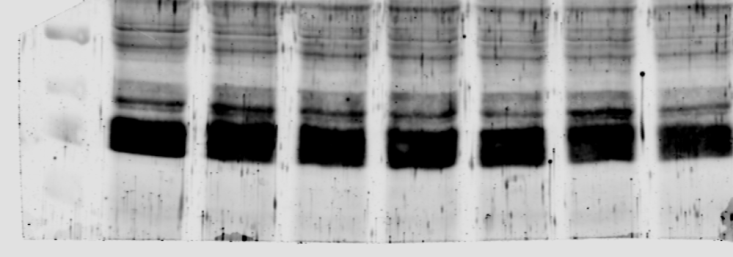

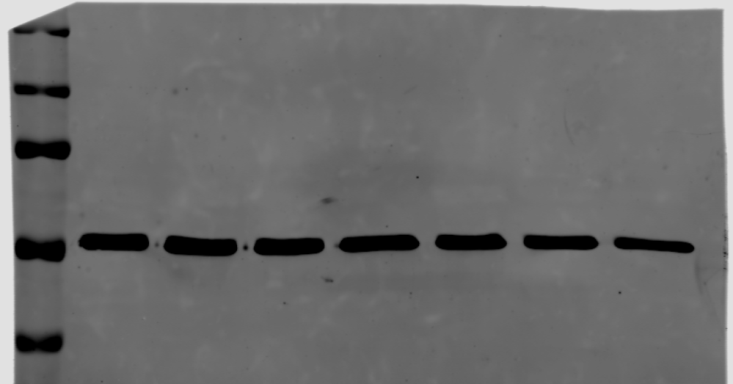


37-

50-

25-

50-

***RPA2***

***α-tub***

**Figure S6. The decrease of CPT-mediated RPA2 hyperphosphorylation is confirmed by immunoblotting analysis.**

HeLa human cervical epithelial carcinoma cells were pre-incubated with the NU7441 or wortmannin (Wortm) inhibitors for 24 h. Then the cells were treated with 10 µM CPT for 1 h, and cellular extracts were analyzed by immunoblotting. Molecular Marker (lane 1), cells no-treated (lane 2), Cells treated with CPT alone (lane 3) or in presence of Nu7441 at 1µM (lane 4), 10µM (lane 5), 100µM (lane6) or Wortmannin at 1µM (lane 7), 10µM (lane 8).

The pre-treatment with the NU7441 inhibitor at 100µM were not shown in the Figure 6 in order to improve the understanding and the comparison with Wortmannin pre-treatment conditions. The blots shown in the Figure 6 were delineated from the full blots.
